# Supplementary material for: Association between Healthy Eating Index and Mental Health in Middle-Aged Adults Based on Household Size in Korea
Source: Int J Environ Res Public Health. 2022 Apr 13;19(8):4692. doi: 10.3390/ijerph19084692 (PMC9032092; doi:10.3390/ijerph19084692)
Supplement: Supplementary file 1 [file ijerph-19-04692-s001.zip › ijerph-1626207-supplementary.pdf]

Table S1 Supplementary. Korean Healthy Eating Index components and standards for scoring

Classification (No. of components)	Components	Score range	Standard for maximum score	Standard for minimum score
Adequacy (8)	Have breakfast*	0-10	5-7 times/w	0 times/w
	Mixed grains intake*,†	0-5	≥ 0.3 serving/d	0 serving/d
	Total fruits intake†	0-5	• Men aged 19-64 years: ≥ 3 serving/d<br>• Men aged 65 years and overs: ≥ 2 serving/d<br>• Women aged 19-64 years: ≥ 2 serving/d<br>• Women aged 65 years and overs: ≥ 1 serving/d	0 serving/d
	Fresh fruits intake*,†	0-5	• Men aged 19-64 years: ≥ 1.5 serving/d<br>• Women aged 19-64 years: ≥ 1 serving/d<br>• Men aged 65 years and overs: ≥ 1 serving/d<br>• Women aged 65 years and over: ≥ 0.5 serving/d	0 serving/d
	Total vegetables intake†	0-5	• Men and women aged 19-64 years: ≥ 8 serving/d<br>• Men aged 65 years and overs: ≥ 8 serving/d<br>• Women aged 65 years and overs: ≥ 6 serving/d	0 serving/d
	Vegetables intake excluding Kimchi and pickled vegetables intake*,†	0-5	• Men and women aged 19-64 years: ≥ 5 serving/d<br>• Men aged 65 years and overs: ≥ 5 serving/d<br>• Women aged 65 years and overs: ≥ 3 serving/d	0 serving/d
	Meat, fish, eggs and beans intake†	0-10	• Men aged 19-64 years: ≥ 5 serving/d<br>• Women aged 19-64 years: ≥ 4 serving/d<br>• Men aged 65 years and overs: ≥ 4 serving/d<br>• Women aged 65 years and overs: ≥ 2.5 serving/d	0 serving/d
	Milk and milk products intake†	0-10	≥ 1 serving/d	0 serving/d
Moderation (3)	Percentage of energy from saturated fatty acid†,‡	0-10	≤ 7% of total energy intake	> 10% of total energy intake
	Sodium intake†,§	0-10	≤ 2,000 mg/d	> 6,500 mg/d
	Percentage of energy from sweets and beverages†	0-10	≤ 10% of total energy intake	> 20% of total energy intake
Balance of energy intake (3)	Percentage of energy from carbohydrate†,§	0-5	55-65% of total energy intake	< 50% or > 75% of total energy intake
	Percentage of energy intake from fat†,‡,§	0-5	15-30% of total energy intake	< 10% or > 35% of total energy intake
	Energy intake†,§	0-5	75-125% of the estimated energy intake requirement (EER)	< 60% or > 140% of EER

* Dietary guidelines for Korean adults

†Dietary Reference Intake for Koreans 2015

‡Recommendation criteria of WHO/FAO

§15 or 85 percentile value in Korean adults aged 19 years and over

From Yook et al. J Nutr Health 2015, 48, 419-428. http://dx.doi.org/10.4163/jnh.2015.48.5.419
